# Redescription of *Crematogaster cypria* Santschi, 1930, new status, with description of two new related species from Greece and Turkey (Hymenoptera, Formicidae)

**DOI:** 10.3897/zookeys.505.9566

**Published:** 2015-05-21

**Authors:** Sebastian Salata, Lech Borowiec

**Affiliations:** 1 Department of Biodiversity and Evolutionary Taxonomy, University of Wrocław, Przybyszewskiego, 63/77, 51-148 Wrocław, Poland

**Keywords:** Mediterranean Subregion, Crematogastrini, Cyprus, Greece, Turkey, taxonomy, *Crematogaster*

## Abstract

Crematogaster (Crematogaster) jehovae var. cypria Santschi, 1930 is raised to species rank. Two new, related species are described from the north-eastern part of the Mediterranean Basin: Crematogaster (Crematogaster) erectepilosa **sp. n.** (Dodecanese, Greece) and Crematogaster (Crematogaster) gullukdagensis **sp. n.** (Antalya Prov., Turkey). These three species are well distinguished from other species of the subgenus *Crematogaster* of the north-eastern part of the Mediterranean Basin in their first gastral tergite bearing numerous erect setae. Colour photographs of all taxa are provided, a key to the species of *Crematogaster cypria* group and species groups of the *Crematogaster* s. str. from the north-eastern Mediterranean region are given and a list of *Crematogaster* s. str. described from this region is provided (see Appendix).

## Introduction

The genus *Crematogaster* Lund, 1831, a member of the subfamily Myrmicinae, is one of the most speciose ant genera. The most recent catalogue lists 487 valid extant species (with fifty-three synonyms, five homonyms and five unavailable names) and 296 valid subspecies, one fossil species was also described (Bolton 2015). Twenty-four species from two subgenera (twenty-two species in *Crematogaster* s. str. and two in *Orthocrema* Santschi) have been so far recorded from Europe and the Mediterranean. In addition, many infraspecific valid names have also been proposed, some of them likely representing distinct taxa whose status needs revision (Borowiec 2014). Mediterranean species of the nominotypical subgenus are mostly similar morphologically and demonstrate a tendency to form local and geographical variations. As a result of that, the differences among species are often not well expressed and correct determination is hindered. Hitherto no key to all European and Mediterranean species has been published, except the outdated key by Santschi (1937), and only local keys exist (Collingwood 1978, Agosti and Collingwood 1987, Cagniant 2005, Seifert 2007, Karaman 2008, Taylor 2010).

During our studies on the ants of Balkans and Cyprus we collected numerous *Crematogaster* samples and concluded that this group is more speciose than local catalogues and keys suggested. We also found some novel characters useful in distinguishing closely related taxa. In this paper we revise a small group of species well distinguished from all taxa of the region by having the first gastral tergite bearing numerous erect setae. This character has never been observed in taxa from the north-eastern part of the Mediterranean Basin but occurs in some North African species, e.g. *Crematogaster oasium* Santschi and some taxa of *Crematogaster laestrygon* complex (our unpublished data).

## Material and methods

Specimens were compared using standard methods of comparative morphology. Photographs were taken using a Nikon SMZ 1500 stereomicroscope, Nikon D5200 photo camera and Helicon Focus software.

All given label data are in their original spelling; a vertical bar (|) separates data on different rows and double vertical bar (||) separates labels. Additional information about the labels or explanatory notes are given in square brackets.

### Abbreviations to collections

CASC California Academy of Sciences, San Francisco, California, USA;

DBET Department of Biodiversity and Evolutionary Taxonomy, University of Wrocław, Poland;

MNHW Museum of Natural History, University of Wrocław, Poland;

SSC Sebastian Salata collection (Wrocław, Poland);

TU Biological Department, Trakya University, Edirne, Turkey.

#### Measurements and indices:

(All lengths are in mm.)

##### Measurements

HL head length; measured in straight line from mid-point of anterior clypeal margin to mid-point of occipital margin; in full face view;

HW head width; measured in full-face view, directly above the eyes;

EL eye length; measured along the maximum diameter of eye;

EW eye width; measured along the maximum width of eye perpendicular to EL;

SL scape length; maximum straight-line length of scape;

PNW pronotum width; maximum width of pronotum in dorsal view;

ML mesosoma length; measured as diagonal length from the anterior end of the neck shield to the posterior margin of the propodeal lobe (equivalent with Weber’s length);

MH mesosoma heigh; measured from the upper edge of mesonotum to the lowest point of the mesopleuron margin; in profile view;

SDL spiracle to declivity length; minimum distance from the center of the propodeal spiracle to the propodeal declivity;

PSL propodeal spine length; measured from the center of the propodeal spiracle to the top of the propodeal spine in lateral view;

PH petiole height; maximum height of petiole in lateral view;

PL petiole length; maximum length of petiole in lateral view;

PW petiole width; maximum width of petiole in dorsal view;

PPH postpetiole height; maximum height of postpetiole in lateral view;

PPL postpetiole length; maximum length of postpetiole in lateral view;

PPW postpetiole width; maximum width of postpetiole in dorsal view;

LHT hind tibia length; maximum length of hind tibia.

Example of measurements: 1.617 ± 0.135 (1.073-1.717) = average measurement ± standard deviation (range of variation).

##### Indices

CI cephalic index: HW/HL × 100;

SI1 scape index 1; SL/HL × 100;

SI2 scape index 2; SL/HW × 100;

MI mesosoma index; ML/PNW × 100;

SPI propodeal spines index; SDL/PSL × 100;

PI1 petiole index 1; PL/PH × 100;

PI2 petiole index 2; PW/PNW × 100;

PI3 petiole index 3; PW/PPW × 100;

PPI1 postpetiole index 1; PPL/PPH × 100;

PPI2 postpetiole index 2; PPW/PNW × 100;

TI hind tibia index 1; LHT/HW × 100;

EI eye index 1; EW/EL × 100;

EI1 eye index 2; EL/HL × 100;

EI2 eye index 3; EW/HL × 100.

## Descriptions

### Crematogaster (Crematogaster) cypria

Taxon classificationAnimaliaHymenopteraFormicidae

Santschi, 1930 new status

**Figures 1–2.**
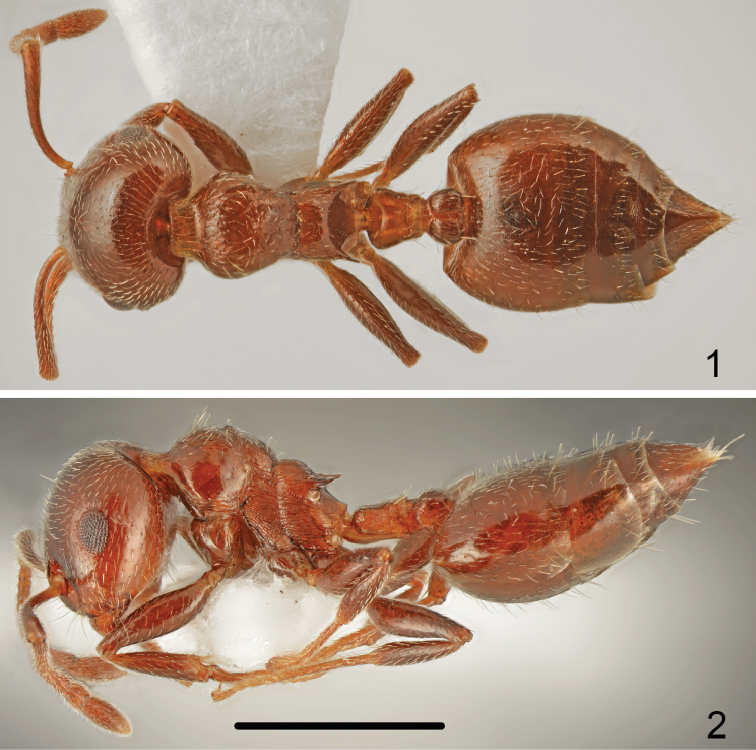
*Crematogaster cypria* Santschi, worker **1** dorsal **2** lateral. Scale bar: 1 mm.

Figs 1 , 2 , 8 , 12 , 15

Crematogaster (Acrocoelia) jehovae For. var. cypria Santschi, 1930: 266.

#### Locus typicus.

Yermasogia river (now Germasogeia [=Yermasoyia] river in Limassol District).

#### Material examined.

Type material: syntype worker on photo (AntWeb resources: Available from: Photo by Alexandra Westrich | URL: http://www.antweb.org/specimen/casent0912688; accessed 18 February 2015): Cr. Jehovae | v Fo | cypria Sant || Chypre | Yermasogia | River. 6.II.30 | G. Mavromoustakis || Type || Sammlung | Dr. F. Santschi | Kairouan || ANTWEB | CASENT | 0912688.

Other material examined: 11 workers – Collection L. Borowiec | Formicidae | LBC-CY00067 || CYPRUS, Paphos distr., 17 m | Avakas Peen., Avakas Gorge | mouth 34.91826 N /32.32978 E | 2 V 2012, L. Borowiec || Crematogaster | cypria | det. L. Borowiec (DBET, CASC); 13 workers – Collection L. Borowiec | Formicidae | LBC-CY00067 || CYPRUS, Paphos distr., 755 m | Panagia-Cedar Valley rd. | 34°55.635 N/32°38.838 E | 5 V 2012, L. Borowiec || Crematogaster | cypria | det. L. Borowiec || (DBET, TU); 2 workers – Collection L. Borowiec | Formicidae | LBC-CY00190 || CYPRUS, Limassol Distr. | Agros, 1062 m | 34.9105 N/33.011 E | 19 VIII 2001, leg. Tsausis || Crematogaster | cypria | det. L. Borowiec (DBET).

#### Differential diagnosis.

*Crematogaster cypria* at first glance is very similar to *Crematogaster jehovae* from the Near East; both species have short propodeal spines and pronotum only dorsolaterally with rugae. However, *Crematogaster cypria* differs in the first gastral tergite bearing numerous erect setae, whereas in *Crematogaster jehovae* the first gastral tergite is bearing appressed hairs, with a row of erect setae only along the posterior margin of the tergite. From *Crematogaster erectepilosa* sp. n. and *Crematogaster gullukdagensis* sp. n. it is easily distinguished by shorter propodeal spines and a shorter mesonotal keel (see the key below). *Crematogaster oasium* Santschi, distributed from Algeria to Saudi Arabia, is a similar species, but differs in having very short propodeal spines forming denticles (in *Crematogaster cypria* the spine is distinct, approximately twice as long as wide at its base) and the dorsum of the pronotum is distinctly dull (shiny in *Crematogaster cypria*).

#### Redescription.

Measurements: Workers (n=24): HL: 0.88 ± 0.048 (0.804-1.017); HW: 0.898 ± 0.062 (0.804-1.061); SL: 0.739 ± 0.025 (0.698-0.816); EL: 0.212 ± 0.014 (0.19-0.251); EW: 0.165 ± 0.01 (0.156-0.19); ML: 1.003 ± 0.066 (0.882-1.212); PSL: 0.146 ± 0.019 (0.112-0.19); SDL: 0.06 ± 0.01 (0.034-0.089); PL: 0.359 ± 0.027 (0.313-0.413); PPL: 0.207 ± 0.017 (0.179-0.24); PH: 0.23 ± 0.018 (0.201-0.268); PPH: 0.259 ± 0.02 (0.215-0.302); PNW: 0.57 ± 0.037 (0.503-0.67); LHT: 0.688 ± 0.029 (0.648-0.771); PW: 0.349 ± 0.04 (0.302-0.436); PPW: 0.302 ± 0.029 (0.263-0.38); CI: 101.8 ± 2.13 (96.6-108.2); SI1: 84.1 ± 2.8 (79.9-89.5); SI2: 82.8 ± 3.5 (74.8-87.6); MI: 175.9 ± 4.8 (160.7-183.4); SPI: 41.0 ± 6.6 (26.6-54.5); PI1: 156.5 ± 8.4 (139.6-172.9); PI2: 61.2 ± 4.6 (55.2-75.6); PPI1: 80.0 ± 4.1 (72.8-86.8); PPI2: 53.0 ± 2.7 (49.6-61.8); HTI: 76.5 ± 2.3 (72.6-80.3); EI: 77.9 ± 3.6 (70.6-85.7); EI1: 24.2 ± 1.0 (23.0-26.0); EI2: 19.1 ± 0.5 (18.3-20.0).

Colour uniformly brown or reddish-brown, mesosoma usually not or only slightly paler coloured than head and abdomen, antennae and legs the same colour as mesosoma (Figs 1, 2).

Head shape quadrate, approximately as wide or slightly wider than long (CI: 101.8 ± 2.13), posterior margin of head in full-face view straight and laterally rounded, occipital carinae distinct (Fig. 8). Antennal scapes reaching or surpassing head margin. Midline of eyes situated slightly above midline of head in full-face view, eyes moderately large (EI1: 24.2 ± 1.0) and slightly protruding. Pronotum laterally rounded, without sharp lateral margins, promesonotal suture indistinct, mesonotum without posterior face, more or less forming one plane with pronotum. Metanotal groove deep, laterally constricted; propodeal spines short, approximately two times as long as wide at base, spiniform, not curved downwards (Fig. 2). Dorsal face of propodeum short but distinct, convex in profile, posterior face of propodeum distinctly sloping, without or with a very shallow transverse groove. Petiole in dorsal view cordiform, dorsum flat, without posterolateral tubercules or denticles, sides carinate, subpetiolar process absent. Postpetiole distinctly bilobed, with a moderately broad median impression, subpostpetiolar process absent.

Head surface finely and sparsely punctate, without microreticulation between punctures, shiny. Masticatory margin of mandibles with four teeth, surface of mandibles distinctly carinate. Clypeus mostly smooth, only on sides with thin, short carinae. Antennal scrobes laterally with 5–7 short carinae not reaching to mid-length of eye. Whole surface of head appears shiny. Vestiture of head mostly with sparse, short, adjacent hairs and 5–8 long erect setae on frons and several long erect setae on underside. Antennal scapes on anterior and dorsal surface covered with suberect setae, on posterior surface basally with adjacent and distally suberect setae (Figs 8, 11). Surface of scape microreticulate. Pronotum only dorsolaterally with short longitudinal rugae, anterior face punctate and microtuberculate at base of setae with very short and sparse carinae, posterior face with slightly carinate setose punctures, sometimes with very thin transverse wrinkles but surface of pronotum appears more or less shiny. Whole dorsal surface of pronotum bearing mixed sparse, short and suberect and long erect setae. Sides of pronotum mostly smooth and shiny with more or less distinct thin, transverse carinae. Mesonotum dorsally in anterior half mostly without sculpture, more or less shiny, without distinct median keel only close to promesonotal suture with small tubercle, in posterior half with thin transverse carinae. Surface of mesonotum with very sparse, short adjacent setae, one to two moderately long, erect setae in anterior part and two pairs of setae posterolaterally. Mesopleuron on whole surface with dense transverse carinae. Dorsal face of propodeum microreticulate, with longitudinal carinae and very sparse and short adjacent pubescence, slope of propodeum smooth and shiny, metapleuron on whole surface with dense, transverse carinae. Petiole on sides with one long and one short erect setae, postpetiolar tubercles with 2-3 erect setae. First gastral tergite with very short and sparse basic pubescence and on whole surface with sparse, moderately long erect setae (Fig. 2), subsequent tergites with row of erect setae along posterior margins. Whole surface of tergites with very fine microreticulation, appears shiny. First sternite with short and sparse basic pubescence and numerous long, erect setae. Legs bearing sparse, short, adjacent pubescence.

#### Distribution.

Known only from Cyprus (Fig. 20).

#### Biological data.

Ants were collected on stems of shrubs, on ground around the shrubs, and on rocks. Locality near Avakas Gorge was located near sea shore, only 17 m a.s.l., in a shallow valley of an intermittent stream. The following ant species were recorded in the same area: *Aphaenogaster sporadis* Santschi, *Camponotus cecconii* Emery, *Lepisiota* sp., Messor cf. structor, *Messor* sp., *Monomorium bicolor* Emery, and *Tapinoma simrothi* Krausse. Locality on roadside between Panagia and Cedar Valley was situated in a montane pine forest at altitude of 755 m. The following ant species were recorded in the same area: *Aphaenogaster sporadis* Santschi, *Camponotus honaziensis* Karaman & Aktaç, *Camponotus jaliensis* Dalla Torre, *Camponotus sanctus* Forel, Cataglyphis cf. nodus, Crematogaster cf. ionia, *Messor wasmanni* Krausse, *Pheidole pallidula* (Nylander), *Plagiolepis taurica* Santschi, Temnothorax cf. recedens, and Tetramorium cf. caespitum.

### Crematogaster erectepilosa sp. n.

Taxon classificationAnimaliaHymenopteraFormicidae

http://zoobank.org/7FB3C7FB-3C73-4B2A-89C4-B8EFED0F155C

**Figures 3–4.**
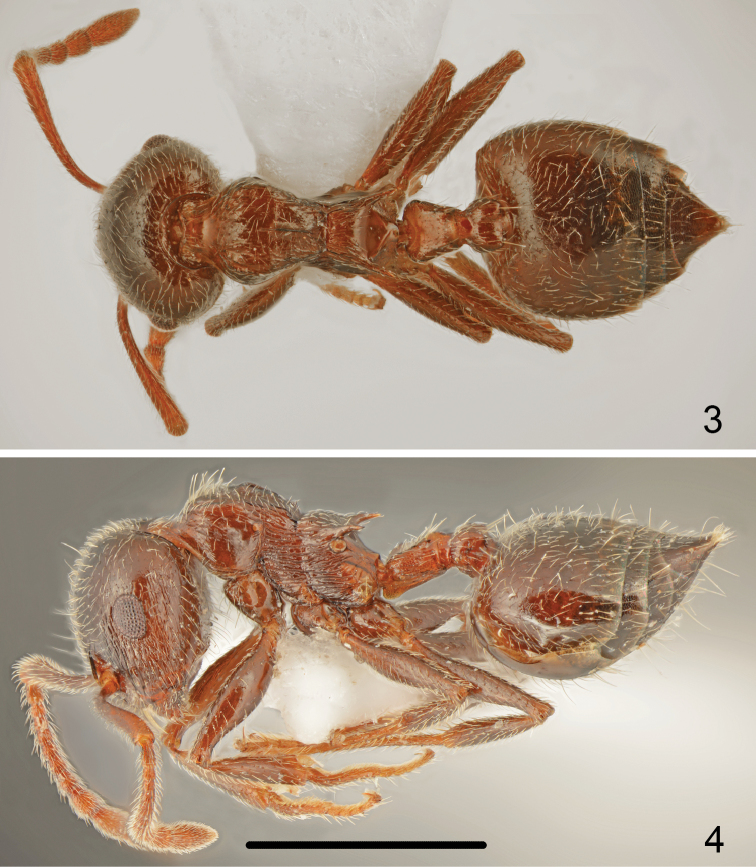
*Crematogaster erectepilosa* sp. n., worker **3** dorsal **4** lateral. Scale bar: 1 mm.

Figs 3 , 4 , 7 , 10 , 14

#### Type material.

Holotype worker – Collection L. Borowiec | Formicidae | LBC-GR01365 || GREECE, Dodecanese | Karpathos, Olympos, 429 m | 35,72448 N/27,1697 E | 19 V 2014, S. Salata (MNHW no. 1222 ); 18 paratype workers: the same data as holotype (DBET, CASC, TU no. ANTWEB1008777-ANTWEB1008794); 2 paratype workers – Collection L. Borowiec | Formicidae | LBC-GR01364 || GREECE, Dodecanese, 385 m | Karpathos, Spoa-Mesochori rd. | loc 2., 35,63108 N/27,13624 E | 22 V 2014, S. Salata (DBET no. ANTWEB1008795-ANTWEB1008796); 22 paratype workers – GREECE, Dodecanese, 385 m | Karpathos, Spoa-Mesochori rd. | loc 2., 35,63108 N/27,13624 E | 22 V 2014, S. Salata (DBET, SSC no. ANTWEB100879-ANTWEB1008818); 1 paratype worker – Collection L. Borowiec | Formicidae | LBC-GR01364 || GREECE, Dodecanese, 399 m | Karpathos, Spoa-Mesochori rd. | 35,62748 N/27,12748 E | 21 V 2014, S. Salata (DBET no. ANTWEB1008819); 1 paratype worker – GREECE Dodecanese | Karpathos, Ag. Nikolaos, | 189 m 35°38'N 27°09'E | 20.05.14 S. Salata (SSC no. ANTWEB1008820); 32 paratype workers – GREECE Karpathos | Trachanammos, 0 m. | 35°27'N 27°06'E | 22.05.14 S. Salata (DBET, SSC no. ANTWEB1008821-ANTWEB1008852); 4 paratype workers – GREECE Dodecanese | Karpathos, Achamandria, | 222 m 35°41'N 27°09'E | 18.05.14 S. Salata (SSC no. ANTWEB1008853-ANTWEB1008856); 1 paratype worker – GREECE Dodecanese | Karpathos, Olympos, 351 m | 35°43'N 27°10'E | 19.05.14 S. Salata (SSC no. ANTWEB1008857); 2 paratype workers – GREECE Dodec. Karpathos, | Vanada, 460 m 35°33' | N/27°09'E, 12.10.2013 | Lymberakis (SSC no. ANTWEB1008858-ANTWEB1008859); 1 paratype worker – GREECE Dodec. Rodos, | Prasonisi, 17 m 36°58' | N/27°44'E, 9.07.2006 | Chatzaki M. (SSC no. ANTWEB1008860); 1 paratype worker – GREECE Dodec. | Kandelioussa, 76 m 36°30'N | /26°58'E, 6.06.2006 | Chatzaki M. (SSC no. ANTWEB1008861); 1 paratype worker – Collection L. Borowiec | Formicidae | LBC-GR01551 || GREECE, Dodecanese, Rodos | Prasonisi, 9 VII 2006, 14 m | 35,8842 N 27,768 E | leg. M. Chatzaki (DBET no. ANTWEB1008862); 1 paratype worker – Collection L. Borowiec | Formicidae | LBC-GR01550 || GREECE, Dodecanese, 270 m | Kos, Pelli | 36,8352/N 27,1668 E | 9 IX 2001 leg. M. Chatzaki (DBET no. ANTWEB1008863);

#### Differential diagnosis.

*Crematogaster erectepilosa* sp. n. differs from all species from the north-eastern part of the Mediterranean Basin, except *Crematogaster cypria* Santschi and *Crematogaster gullukdagensis* sp. n., in that the first gastral tergite bearing numerous erect setae. *Crematogaster cypria* is well distinguished by shorter propodeal spines and mesonotal keel (see key below). *Crematogaster gullukdagensis* is very similar but differs in having the antennal scape predominantly with subappressed and suberect setae (Fig. 11), while in *Crematogaster erectepilosa* sp. n. the setae on scape are mostly erect (Fig. 10). Head in full face view in *Crematogaster erectepilosa* sp. n. appears round, while in *Crematogaster gullukdagensis* sp. n. it is slightly square. Eyes in *Crematogaster erectepilosa* sp. n. are more round (EI 74.3 ± 2.3 [71.5-78.8]) and in *Crematogaster gullukdagensis* sp. n. they are more oval (EI 69.5 ± 3.1 [63.4-73.6]). Body ground colour in *Crematogaster erectepilosa* sp. n. is darker, yellowish-brown to brown, in *Crematogaster gullukdagensis* sp. n. yellowish to pale yellowish-brown. Propodeal spines of *Crematogaster erectepilosa* sp. n. in most specimens are slightly curved down, while in *Crematogaster gullukdagensis* sp. n. propodeal spines are mostly straight, spine at base slightly thicker in *Crematogaster erectepilosa* sp. n. and thinner in *Crematogaster gullukdagensis* sp. n. Sides of pronotum in *Crematogaster erectepilosa* sp. n. in most specimens have fine longitudinal striation, while in *Crematogaster gullukdagensis* sp. n. they are mostly without striation, smooth and shiny.

#### Description.

Measurements: Workers (n=23): HL: 0.948 ± 0.039 (0.872-1.017); HW: 0.972 ± 0.056 (0.872-1.072); SL: 0.884 ± 0.027 (0.835-0.921); EL: 0.228 ± 0.007 (0.212-0.235); EW: 0.169 ± 0.005 (0.162-0.179); ML:1.117 ± 0.057 (1.011-1.209); PSL: 0.2 ± 0.023 (0.156-0.251); SDL: 0.065 ± 0.03 (0.044-0.165); PL: 0.415 ± 0.014 (0.391-0.436); PPL: 0.207 ± 0.012 (0.19-0.235); PH: 0.228 ± 0.016 (0.19-0.246); PPH: 0.26 ± 0.017 (0.223-0.291); PNW: 0.6 ± 0.03 (0.547-0.654); LHT: 0.81 ± 0.027 (0.777-0.865); PW: 0.337 ± 0.03 (0.236-0.38); PPW: 0.297 ± 0.018 (0.268-0.335); CI: 102.5 ± 1.9 (99.3-105.4); SI1: 93.3 ± 1.9 (89.3-96.0); SI2: 91.1 ± 3.0 (84.7-96.0); MI: 186.1 ± 3.7 (179.6-194.0); SPI: 28.9 ± 4.3 (23.2-37.8); PI1: 184.3 ± 11.7 (167.9-205.8); PI2: 56.2 ± 4.0 (42.2-59.2); PPI1: 79.1 ± 3.5 (73.4-83.7); PPI2: 49.5 ± 1.0 (47.9-51.2); HTI: 84.8 ± 2.6 (81.1-90.4); EI: 74.3 ± 2.3 (71.5-78.8); EI1: 24.1 ± 0.7 (22.7-25.0); EI2: 17.9 ± 0.7 (16.9-18.7).

Colour uniformly pale to dark brown, mesosoma not paler coloured than head and abdomen, legs the same colour, antennae only slightly paler coloured than mesosoma (Figs 3, 4).

Head shape almost round, approximately as wide as long (CI: 102.5 ± 1.9), posterior margin of head in full-face view straight and laterally rounded, occipital carinae distinct (Fig. 7). Antennal scapes slightly surpassing head margin. Midline of eyes situated slightly above midline of head in full-face view, eyes moderately large (EI1: 24.1 ± 0.7) and protruding. Pronotum laterally rounded, with sharp lateral margins, promesonotal suture absent, mesonotum without posterior face more or less forming one plane with pronotum. Metanotal groove deep, laterally constricted; propodeal spines long, 2.7–2.8 times as long as wide at base, spiniform, in most specimens slightly curved downwards (Fig. 4). Dorsal face of propodeum short but distinct, convex in profile, posterior face of propodeum distinctly sloping, without transverse groove. Petiole in dorsal view cordiform, dorsum flat or slightly concave, without posterolateral tubercules or denticles, sides carinate, subpetiolar process absent. Postpetiole distinctly bilobed, with a narrow median impression, subpostpetiolar process absent.

Head surface finely and sparsely punctate, without microreticulation between punctures, shiny. Masticatory margin of mandibles with four teeth, surface of mandibles distinctly carinate. Clypeus on whole surface with thin carinae or only in the middle carinae indistinct. Antennal scrobes laterally with 7–9 long carinae extending to mid length of eye, also genae with carinae and area behind eyes with thin carinae. Whole surface of head appears shiny. Vestiture of head mostly with sparse, short, suberect hairs and 5–8 long erect setae on frons and several long erect setae on underside. Antennal scapes on anterior and dorsal surface bearing long erect setae, on posterior surface basally with suberect and distally erect setae (Figs 7, 10). Surface of scape with indistinct microreticulation, shiny. Pronotum in anterior half and dorsolaterally with longitudinal rugae, posterior face with punctuation and sparse, very short carinae, surface of pronotum appears more or less shiny. Whole dorsal surface of pronotum bearing mixed sparse, short suberect and long erect setae. Sides of pronotum with more or less distinct thin, transverse carinae disappearing from anterior to posterior margin of pronotum but in most specimens well visible. Mesonotum dorsally on whole length with longitudinal and oblique rugae, more or less shiny, with distinct median keel in most specimens running from anterior margin of mesonotum to its ¾ length, in some specimens reaching to posterior margin of mesonotum. Surface of mesonotum with very sparse, short adjacent setae. Mesopleuron on whole surface with dense, transverse carinae. Dorsal face of propodeum with longitudinal carinae and very sparse and short adjacent pubescence, slope of propodeum smooth and shiny, metapleuron on whole surface with dense, transverse carinae. Petiole on sides and posterior half with long erect setae, also postpetiolar tubercles several erect setae. First gastral tergite with sparse, moderately long, suberect basic pubescence and on whole surface with sparse, moderately long erect setae (Fig. 2), subsequent tergites with row of erect setae along posterior margins. Whole surface of tergites with very fine microreticulation, appears shiny. First sternite with moderately long and sparse basic pubescence and numerous long, erect setae. Legs bearing sparse, moderately long, more or less erect pubescence.

#### Etymology.

Named after erect setae on antennal scape.

#### Distribution.

Dodecanese Archipelago in Aegean Greece (Fig. 20).

#### Biological data.

The ants were collected on ground around shrubs and from shrub leaves and stems. Locality on Karpathos, Olympos was placed 429 m a.s.l. in dry, stony and rocky area with sparse shrubs. The following ant species were recorded in the same area: *Aphaenogaster olympica* Borowiec & Salata, *Camponotus gestroi* Emery, *Camponotus honaziensis* Karaman & Aktaç, *Camponotus ionius* Emery, *Camponotus jaliensis* Dalla Torre, *Camponotus kiesenwetteri* (Roger), *Crematogaster ionia* Forel, *Crematogaster sordidula* (Nylander), *Lepisiota nigra* (Dalla Torre), *Messor orientalis* (Emery), *Messor wasmanni* Krausse, *Pheidole pallidula* (Nylander), *Plagiolepis pallescens* sensu Radchenko, *Tapinoma simrothi* Krausse, *Temnothorax exilis* (Emery), *Temnothorax recedens* (Nylander), and *Temnothorax solerii* (Menozzi). First locality on Spoa-Mesochori rd. was on a rocky slope, above olive orchard, overgrown by shrubs. The following ant species were recorded in the same area: *Aphaenogaster karpathica* Boer, *Aphaenogaster olympica* Borowiec & Salata, *Camponotus ionius* Emery, *Camponotus jaliensis* Dalla Torre, *Camponotus kiesenwetteri* (Roger), *Camponotus lateralis* (Olivier), *Crematogaster sordidula* (Nylander), *Lepisiota nigra* (Dalla Torre), *Messor wasmanni* Krausse, *Pheidole pallidula* (Nylander), *Plagiolepis pallescens* sensu Radchenko, *Plagiolepis taurica* Santschi, *Tapinoma simrothi* Krausse, *Temnothorax exilis* (Emery), *Temnothorax semiruber* (André), and Tetramorium cf. punctatum. Second locality on Spoa-Mesochori rd. was near a road, opposite the Spoa-Mesochori rd. locality, area was overgrown by Mediterranean shrubland. The following ant species were recorded in the same area: *Camponotus jaliensis* Dalla Torre, *Camponotus kiesenwetteri* (Roger), *Camponotus lateralis* (Olivier), *Crematogaster ionia* Forel, *Pheidole pallidula* (Nylander), *Plagiolepis pallescens* sensu Radchenko, and *Temnothorax exilis* (Emery).

Locality near Agios Nikolaos was located above the village. The vegetation at this locality is a Mediterranean shrubland and pine forest. The following ant species were recorded in the same area: *Camponotus ionius* Emery, *Camponotus kiesenwetteri* (Roger), *Lepisiota melas* (Emery), *Pheidole pallidula* (Nylander), *Plagiolepis pallescens* sensu Radchenko, Tetramorium cf. caespitum, and Tetramorium cf. punctatum.

Locality near Achamandria was on a dry slope overgrown by Mediterranean shrubland and isolated pine trees. The following ant species were recorded in the same area: *Camponotus gestroi* Emery, *Camponotus ionius* Emery, *Camponotus jaliensis* Dalla Torre, *Camponotus kiesenwetteri* (Roger), *Camponotus lateralis* (Olivier), *Crematogaster ionia* Forel, *Crematogaster sordidula* (Nylander), *Lepisiota nigra* (Dalla Torre), *Pheidole pallidula* (Nylander), *Plagiolepis taurica* Santschi, *Temnothorax exilis* (Emery), *Temnothorax recedens* (Nylander), *Temnothorax semiruber* (André), and *Temnothorax solerii* (Menozzi). Locality near Trachanammos was in a sandy valley created by intermittent river, overgrown by Mediterranean shrubland. Nest was located in the soil, under stone beneath shrubs. The following ant species were recorded in the same area: *Camponotus kiesenwetteri* (Roger), *Lepisiota nigra* (Dalla Torre), *Monomorium subopacum* (F. Smith) and *Pheidole pallidula* (Nylander).

### Crematogaster gullukdagensis sp. n.

Taxon classificationAnimaliaHymenopteraFormicidae

http://zoobank.org/9C76B398-7D55-4039-B93A-7E62C222248B

#### Type material.

Holotype worker: Collection L. Borowiec | Formicidae | LBC-TR00073 || TURKEY, Antalaya Prov. | ancient Termessos | 1018 m, 36°58/30°27 | 3 VII 2010, L. Borowiec (MNHW no. 1223); 15 paratype workers: the same data as holotype (DBET, CASC, TU no. ANTWEB1008863-ANTWEB1008878).

#### Differential diagnosis.

See diagnosis for *Crematogaster erectepilosa* sp. n.

#### Description.

Measurements: Workers (n=16): HL: 0.981 ± 0.024 (0.932-1.027); HW: 1.001 ± 0.041 (0.949-1.084); SL: 0.894 ± 0.033 (0.843-0.988); EL: 0.224 ± 0.011 (0.201-0.246); EW: 0.156 ± 0.004 (0.151-0.168); ML:1.165 ± 0.054 (1.084-1.309); PSL: 0.229 ± 0.024 (0.19-0.294); SDL: 0.06 ± 0.01 (0.044-0.086); PL: 0.464 ± 0.038 (0.424-0.576); PPL: 0.237 ± 0.025 (0.212-0.317); PH: 0.25 ± 0.024 (0.223-0.323); PPH: 0.283 ± 0.019 (0.263-0.338); PNW: 0.618 ± 0.02 (0.575-0.654); LHT: 0.806 ± 0.028 (0.749-0.86); PW: 0.362 ± 0.01 (0.344-0.38); PPW: 0.31 ± 0.015 (0.268-0.335); CI: 101.9 ± 2.0 (99.4-105.6); SI1: 90.7 ± 1.3 (88.7-93.6); SI2: 88.9 ± 1.8 (84.9-91.0); MI: 187.1 ± 4.1 (179.8-191.8); SPI: 26.3 ± 2.8 (20.2-30.4); PI1: 185.4 ± 12.9 (173.7-222.9); PI2: 58.7 ± 1.0 (57.4-60.8); PPI1: 83.9 ± 4.2 (77.8-93.8); PPI2: 50.2 ± 1.9 (43.6-52.5); HTI: 80.8 ± 1.6 (78.9-83.8); EI: 69.5 ± 3.1 (63.4-73.6); EI1: 22.9 ± 1.0 (20.2-24.5); EI2: 16.0 ± 0.3 (15.5-16.7).

**Figures 5–6.**
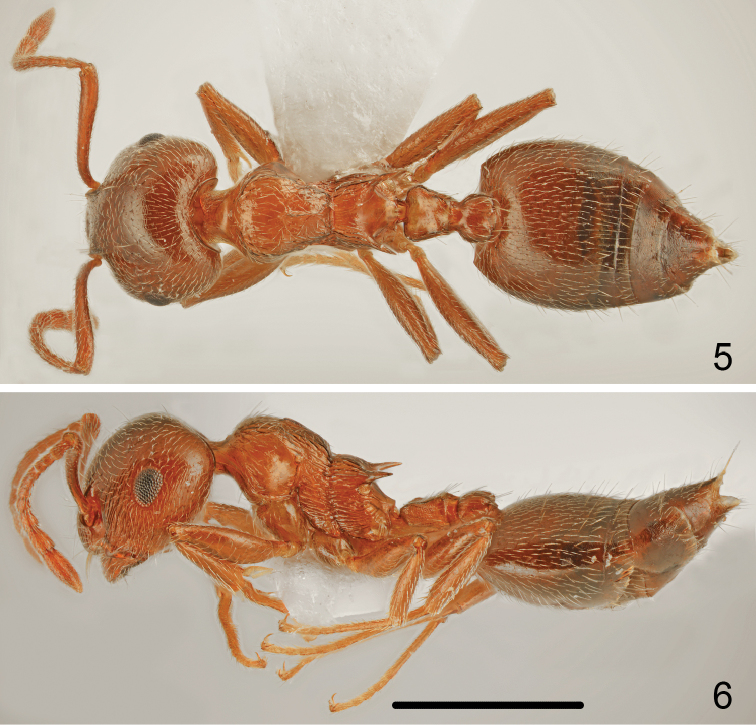
*Crematogaster gullukdagensis* sp. n., worker **5** dorsal **6** lateral. Scale bar: 1 mm.

Colour uniformly yellowish brown to pale brown, mesosoma not paler coloured than head and abdomen, legs and antennae the same colour as mesosoma (Figs 5, 6).

Head shape almost square, approximately as wide as long (CI: 101.9 ± 2.0), posterior margin of head in full-face view straight and laterally rounded, occipital carinae distinct (Fig. 9). Antennal scapes slightly surpassing head margin. Midline of eyes situated slightly above midline of head in full-face view, eyes moderately large (EI1: 22.9 ± 1.0) and protruding. Pronotum laterally rounded, with sharp lateral margins, promesonotal suture absent, mesonotum without posterior face more or less forming one plane with pronotum. Metanotal groove deep, laterally constricted; propodeal spines long, 2.7–2.9 times as long as wide at base, spiniform, in most specimens straight (Fig. 6). Dorsal face of propodeum short but distinct, convex in profile, posterior face of propodeum distinctly sloping, without transverse groove. Petiole in dorsal view cordiform, dorsum flat or slightly concave, without posterolateral tubercules or denticles, sides carinate, subpetiolar process absent. Postpetiole distinctly bilobed, with a narrow median impression, subpostpetiolar process absent.

**Figures 7–8.**
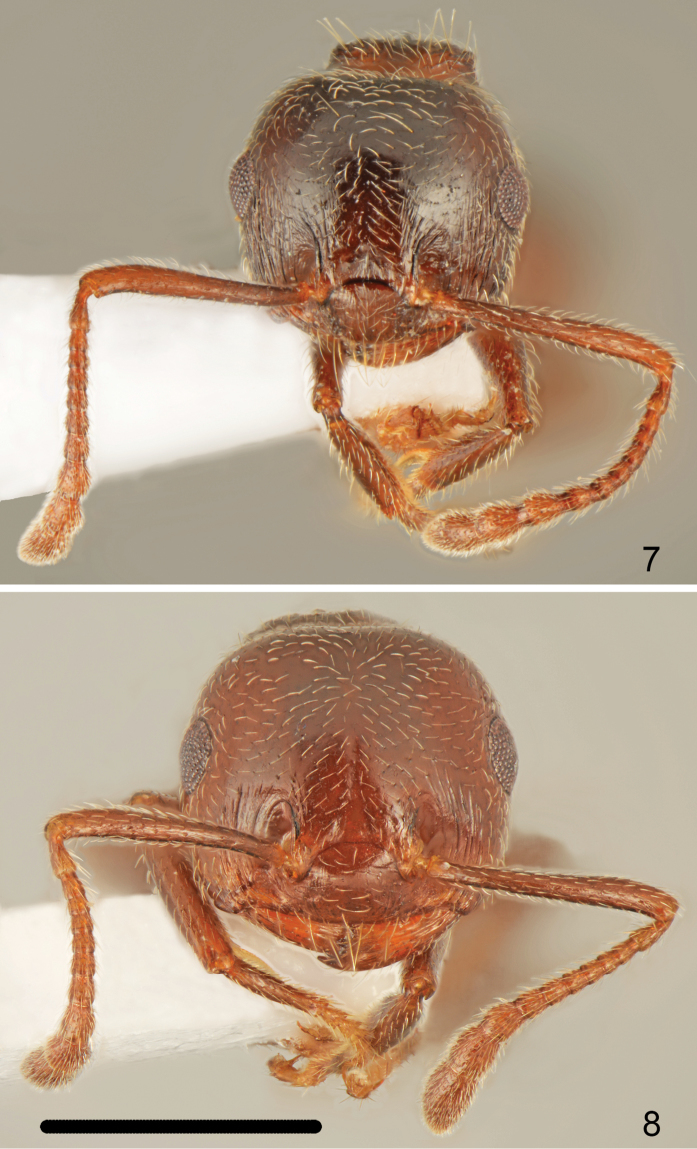
Worker head **7** *Crematogaster erectepilosa* sp. n. **8** *Crematogaster cypria*. Scale bar: 1 mm.

**Figures 9–12.**
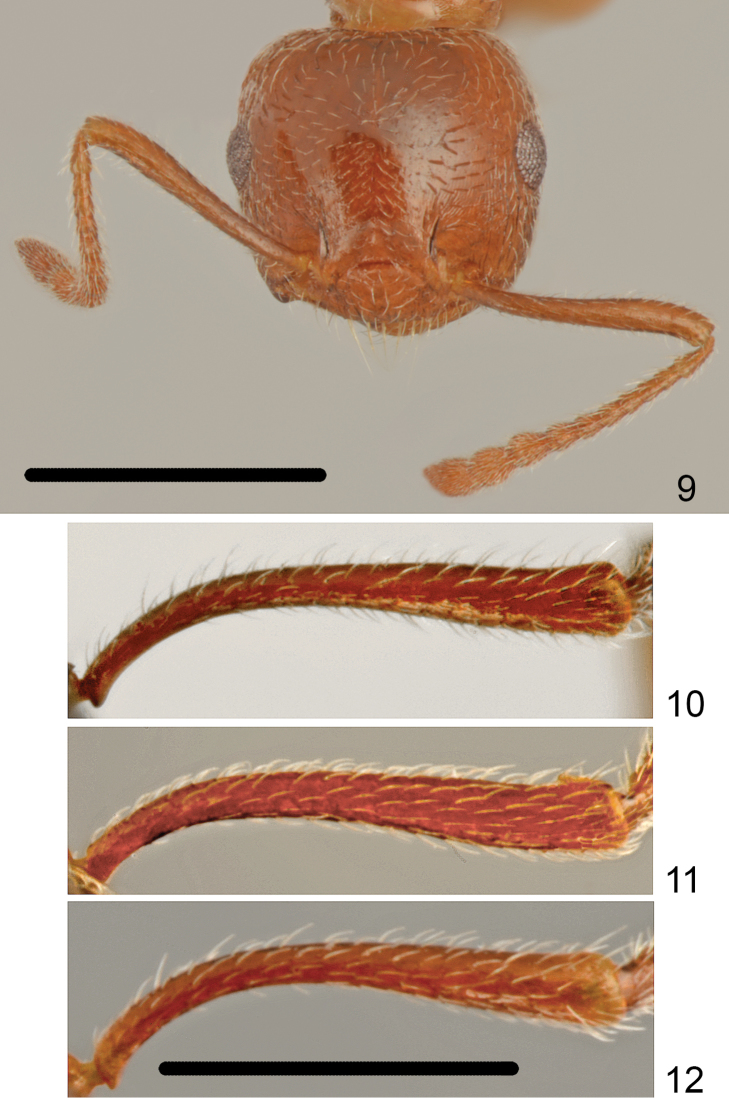
Worker head and scapus **9** *Crematogaster gullukdagensis* sp. n. **10** *Crematogaster erectepilosa* sp. n. **11** *Crematogaster gullukdagensis* sp. n. **12** *Crematogaster cypria*. Scale bar: 1 mm (**9**), 0.5 mm (**10–12**).

**Figures 13–19.**
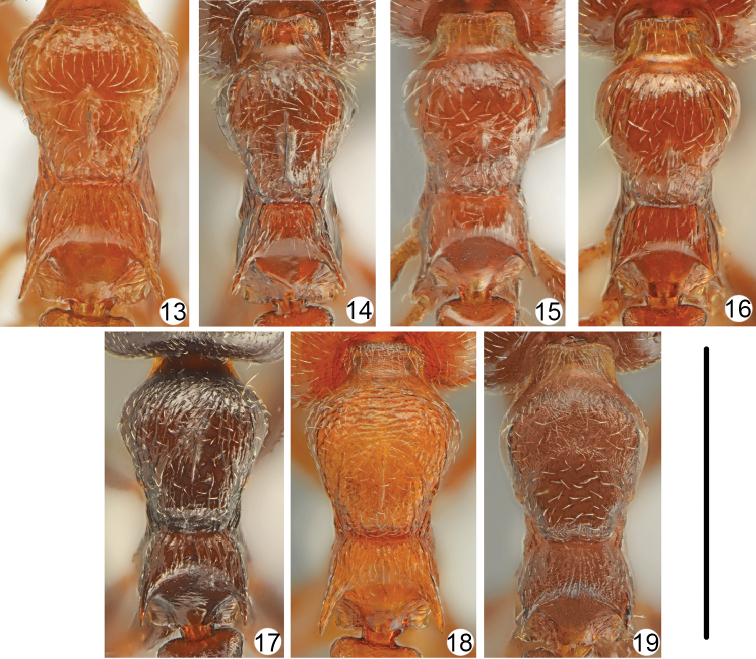
Mesosoma **13** *Crematogaster gullukdagensis* sp. n. **14** *Crematogaster erectepilosa* sp. n. **15** *Crematogaster cypria* **16** *Crematogaster jehovae* **17** *Crematogaster ionia* **18** *Crematogaster schmidti* **19** *Crematogaster lorteti*. Scale bar: 1 mm.

Head surface finely and sparsely punctate, without microreticulation between punctures, shiny. Masticatory margin of mandibles with four teeth, surface of mandibles distinctly carinate. Clypeus laterally with thin carinae, in the middle smooth or with indistinct carinae. Antennal scrobes laterally with 7–9 long carinae extending to mid length of eye, also genae with carinae and area behind eyes with thin carinae. Whole surface of head appears shiny. Vestiture of head mostly with sparse, short, adjacent to suberect hairs and 4–6 long erect setae on frons and several long erect setae on underside. Antennal scapes on anterior and dorsal surface bearing suberect setae, sometimes with 2–3 longer and more erect setae, on posterior surface basally with adjacent and distally suberect setae (Figs 9, 12). Surface of scape with indistinct microreticulation, shiny. Pronotum dorsolaterally with longitudinal rugae, anterior face mostly sparsely punctate and at most with few very short rugae, posterior face only with punctuation, surface of pronotum appears more or less shiny. Whole dorsal surface of pronotum bearing mixed sparse, short adjacent to suberect and long erect setae. Sides of pronotum only in anterior half with more or less distinct thin, transverse carinae, posterior half in most specimens completely smooth. Mesonotum dorsally on sides with longitudinal and oblique rugae, centrally partly smooth, more or less shiny, with distinct median keel in most specimens running from anterior margin of mesonotum to its ½-⅔ length, never reaching to posterior margin of mesonotum. Surface of mesonotum with very sparse, short adjacent setae. Mesopleuron on whole surface with dense, transverse carinae. Dorsal face of propodeum laterally with longitudinal carinae, in central part more or less smooth, with very sparse and short adjacent pubescence, slope of propodeum smooth and shiny, metapleuron on whole surface with dense, transverse carinae. Petiole on sides and posterior half with long erect setae, also postpetiolar tubercles several erect setae. First gastral tergite with sparse, moderately long, adjacent to suberect basic pubescence and on whole surface with sparse, moderately long erect setae (Fig. 6), subsequent tergites with row of erect setae along posterior margins. Whole surface of tergites with very fine microreticulation, appears shiny. First sternite with moderately long and sparse basic pubescence and numerous long, erect setae. Legs bearing sparse, moderately long, adjacent to suberect pubescence.

#### Etymology.

Named after terra typica: Güllük Dag mountains in Antalya Province of Turkey.

#### Distribution.

**Figure 20.**
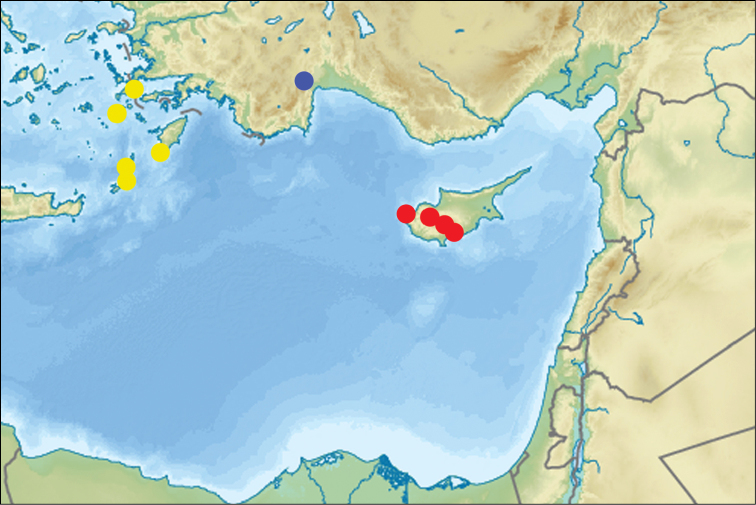
Distribution of *Crematogaster cypria* Santschi (red circles), *Crematogaster erectepilosa* sp. n. (yellow circles) and *Crematogaster gullukdagensis* sp. n. (blue circle).

SW Turkey (Fig. 20).

#### Biological data.

The ants were collected on the trunk of a small oak species and on ground around the tree. The type locality is in a montane area within the ancient Termessos city, at 1018 m a.s.l. The following ant species were recorded from the same area: *Aphaenogaster festae* Emery, *Aphaenogaster maculifrons* Kiran & Aktaç, *Aphaenogaster sporadis* Santschi, *Camponotus aethiops* (Latreille), *Camponotus boghossiani* Forel, *Camponotus lateralis* (Olivier), *Camponotus samius* Forel, *Cataglyphis* sp., Crematogaster cf. ionia, *Lasius lasoides* (Emery), Messor cf. structor, *Pheidole pallidula* (Nylander), *Tetramorium anatolicum* Csösz & Schulz, and Tetramorium cf. semilaeve.

### Key to *Crematogaster* workers from the north-eastern part of the Mediterranean Basin

**Table:** 

1	Petiole subquadrate in dorsal view, sides almost parallel; antennal club three-segmented, sgen. *Crematogaster* s. str.	**2**
–	Petiole trapezoidal narrowing from front to rear in dorsal view, sides almost parallel; antennal club two-segmented, sgen. *Orthocrema*	
2	Propodeum with distinct propodeal spines	**3**
–	Propodeum without propodeal spines, at most with small tubercles. Cyprus, Caucasian countries, the Near East and North Africa	*Crematogaster inermis* complex*
3	First gastral tergite with numerous erect setae (Figs 2, 4, 6)	**4**
–	First gastral tergite without or at most with 1–5 erect setae	**6**
4	Propodeal spines long, more than 2.5 times longer than width at base. Mesonotal keel long, longer than half length of mesonotum (Figs 4, 6)	**5**
–	Propodeal spines short, at most 2 times longer than width at base (Fig. 2). Mesonotal keel absent or forming very small tubercle close to promesonotal suture (Fig. 15). Cyprus	***Crematogaster cypria* Santschi**
5	Antennal scape on anterior surface on whole length with erect setae (Fig. 10). Eyes more round. Dodecanese	***Crematogaster erectepilosa* sp. n.**
–	Antennal scape on anterior surface with subappressed to suberect setae (Fig. 12). Eyes more oval. SW Turkey	***Crematogaster gullukdagensis* sp. n.**
6	Pronotum at least on sides with more or less distinct rugae, dorsal surface more or less shiny (Figs 16–18). Mesonotal keel present, at least in form of short longitudinal tubercle (Figs 16–18)	**7**
–	Pronotum without rugae, dorsal surface punctate and microreticulate, dull. Mesonotal keel absent (Fig. 19). Widespread throughout the region	***Crematogaster lorteti* Forel**
7	Pronotum on whole surface with rugae (Figs 17, 18). Propodeal spines long, more than 2.5 times longer than width at base	**8**
–	Pronotum only on sides with short rugae, anterior and central part only punctate (Fig. 16). Propodeal spines short, at most 2 times longer than width at base. The Near East (Egypt, Israel, Iraq, Jordan)	*Crematogaster jehovae* complex*
8	Body distinctly bicoloured, head and mesosoma yellowish, red to reddish-brown, abdomen dark brown. Rugae on anterior part of pronotum usually transverse (Fig. 18). Widespread throughout the region	*Crematogaster schmidti* complex*
–	Body more or less unicolours, brown to almost black or head and mesosoma only indistinctly paler coloured than abdomen. Rugae on whole pronotum usually longitudinal or on pronotal sides oblique, occasionally in anterior part transverse (Fig. 17). Widespread throughout the region	*Crematogaster ionia* complex*

* These complexes comprise more than one species, some of them probably have been described under valid specific and infraspecific names and some are new to science; all complexes need a revision based on types and material encompassing the entire distribution of these species.

## Supplementary Material

XML Treatment for Crematogaster (Crematogaster) cypria

XML Treatment for Crematogaster erectepilosa

XML Treatment for Crematogaster gullukdagensis

## Appendix

List of *Crematogaster* s. str. taxa described from the north-eastern Mediterranean region

*Crematogaster auberti laestrigon cretica* Karavaiev, 1927 unavailable name

Crematogaster (Acrocoelia) auberti subsp. laestrigon var. cretica Karavaiev, 1927: 106, fig. 2 (w.)

*Crematogaster cypria* Santschi, 1930

Crematogaster jehovae var. cypria Santschi, 1930: 266 (w.)

*Crematogaster gordani* Karaman, 2008

*Crematogaster gordani* Karaman, 2008: 6, figs 1–8, pl. 1.

*Crematogaster inermis aphrodite* Santschi, 1937

Crematogaster inermis var. aphrodite Santschi, 1937: 298, figs 2, 17 (w.q.m.)

*Crematogaster ionia* Forel, 1911

Crematogaster scutellaris var. ionia Forel, 1911: 340 (w.q.)

*Crematogaster lorteti* Forel, 1910

*Crematogaster lorteti* Forel, 1910: 435 (w.q.)

*Crematogaster lorteti hellenica* Forel, 1911

Crematogaster (Atopogyne) hellenica Forel, 1911: 342 (q.)

*Crematogaster montenigrina* Karaman, 2008

*Crematogaster montenigrinus* Karaman, 2008: 14, figs 13–16, pl.1.

Crematogaster scutellaris subsp. schmidti var. atratula Zimmermann, 1935: 21 unavailable name

*Crematogaster phoenica* Santschi, 1915

Crematogaster laestrygon st. phoenica Santschi, 1915: 59 (w.)

*Crematogaster phoenica pygmalion* Santschi, 1934

*Crematogaster phoenica pygmalion* Santschi, 1934: 276 (w.)
